# CHANGES IN THE AGING POLICY LANDSCAPE SINCE 2020: HITS AND MISSES

**DOI:** 10.1093/geroni/igae098.0309

**Published:** 2024-12-31

**Authors:** Debra Lipson, Christine Bishop

**Affiliations:** Mathematica, Washington, District of Columbia, United States; Brandeis University, Waltham, Massachusetts, United States

## Abstract

Despite deep political divisions after the 2020 election, Congress passed several major statutes to address the acute economic and health fallout of the COVID-19 pandemic, including provisions that helped older adults cover large and unexpected health care costs and expanded access to home and community-based care (HCBS). States used federal funds disbursed under these statutes to strengthen the long-term services and supports (LTSS) system and respond to the COVID-19 health emergency. But as the pandemic faded into the distance, so did the impetus to address the fundamental problems that pose the greatest long-term risk to the health and economic security of older adults. This paper reviews major legislative and regulatory initiatives adopted at the federal and state level since 2020 to improve the economic security of low-income older adults, expand Medicare and Medicaid coverage, strengthen nursing home quality and the direct care workforce, increase access to HCBS, and provide greater support to family caregivers. Despite these achievements, progress has been incremental. Little has been done to fix flaws in Medicare and Social Security financing, and policymakers have yet to address LTSS financing, all of which pose major threats to the long-term financial wellbeing of older adults and their families. These issues belong at the top of the aging policy agenda and urgently call for action by the next Administration, Congress, and state and local governments.

